# Predictive Markers of Post-Stroke Cognitive Recovery and Depression in Ischemic Stroke Patients: A 6-Month Longitudinal Study

**DOI:** 10.3390/ejihpe14120200

**Published:** 2024-12-11

**Authors:** Anna Tsiakiri, Spyridon Plakias, Pinelopi Vlotinou, Aikaterini Terzoudi, Aspasia Serdari, Dimitrios Tsiptsios, Georgia Karakitsiou, Evlampia Psatha, Sofia Kitmeridou, Efstratios Karavasilis, Nikolaos Aggelousis, Konstantinos Vadikolias, Foteini Christidi

**Affiliations:** 1 Department of Neurology, Democritus University of Thrace, 68100 Alexandroupolis, Greece; terzoudi@med.duth.gr (A.T.); dtsipt@auth.gr (D.T.); s.kitmer@gmail.com (S.K.); kvadikol@med.duth.gr (K.V.); christidi.f.a@gmail.com (F.C.); 2 Department of Physical Education and Sport Science, University of Thessaly, 42100 Trikala, Greece; spyros_plakias@yahoo.gr; 3 Department of Occupational Therapy, University of West Attica, 12243 Athens, Greece; pvlotinou@uniwa.gr; 4 Department of Child and Adolescent, Democritus University of Thrace, 68100 Alexandroupolis, Greece; aserntar@med.duth.gr; 5 3rd Department of Neurology, Aristotle University of Thessaloniki, 54124 Thessaloniki, Greece; 6 Department of Psychiatry, Medical School, Democritus University of Thrace, 68100 Alexandroupolis, Greece; gkarakit@med.duth.gr; 7 Department of Radiology, School of Medicine, Democritus University of Thrace, 68100 Alexandroupolis, Greece; epsatha@med.duth.gr; 8 Medical Physics Laboratory, Democritus University of Thrace, 68100 Alexandroupolis, Greece; ekaravas@med.duth.gr; 9 Department of Physical Education and Sport Science, Democritus University of Thrace, 69100 Komotini, Greece; nagelous@phyed.duth.gr

**Keywords:** ischemic stroke, predictive markers, demographics, stroke severity, functional status, neuropsychological assessment, cognitive recovery, depression

## Abstract

The growing number of stroke survivors face physical, cognitive, and psychosocial impairments, making stroke a significant contributor to global disability. Various factors have been identified as key predictors of post-stroke outcomes. The aim of this study was to develop a standardized predictive model that integrates various demographic and clinical factors to better predict post-stroke cognitive recovery and depression in patients with ischemic stroke (IS). We included IS patients during both the acute phase and six months post-stroke and considered neuropsychological measures (screening scales, individual tests, functional cognitive scales), stroke severity and laterality, as well as functional disability measures. The study identified several key predictors of post-stroke cognitive recovery and depression in IS patients. Higher education and younger age were associated with better cognitive recovery. Lower stroke severity, indicated by lower National Institutes of Health Stroke Scale (NIHSS) scores, also contributed to better cognitive outcomes. Patients with lower modified Rankin Scale (mRS) scores showed improved performance on cognitive tests and lower post-stroke depression scores. The study concluded that age, education, stroke severity and functional status are the most critical predictors of cognitive recovery and post-stroke emotional status in IS patients. Tailoring rehabilitation strategies based on these predictive markers can significantly improve patient outcomes.

## 1. Introduction

Stroke is a leading cause of morbidity and mortality worldwide, with its impact on public health being substantial. Of note, ischemic stroke (IS) accounts for approximately 80% of all strokes. While mortality rates have slowly declined over recent years, a growing number of stroke survivors are left to cope with a wide range of physical, cognitive, functional, and psychosocial impairments [1]. The latter yields stroke as one of the most significant contributors to global disability, with several factors, such as increasing age, cardiovascular risks, low education levels, and the severity of the stroke, identified as strong predictors of post-stroke outcomes [2]. These determinants, in combination with pre-existing conditions like atherosclerotic disease and previous strokes, have been consistently linked to worse recovery and long-term prognosis [3,4,5,6]. The latter is thus a multifaceted process influenced by various patient-specific and stroke-related factors, making it challenging to predict outcomes and responses to rehabilitation.

Post-stroke cognitive impairment (PSCI) encompasses a variety of deficits, particularly in executive function, memory, attention, and language. Cognitive dysfunction can affect 31% to 77% of stroke patients, depending on stroke severity and demographic factors [7,8]. Acute phase cognitive impairments can be present in up to 90% of patients [9,10], with improvement seen in the first three to six months [10]. However, cognitive deficits can persist even after this period, with 30% of survivors continuing to show impairments at three months, and up to 42% at five years post-stroke [11]. Chronic impairments, particularly in executive functions, significantly contribute to long-term disability and dependency. Identifying predictors like age, education, stroke severity, and lesion location early on is critical, as these factors influence recovery trajectories. Imaging markers such as infarct volume and white matter changes also play a role in determining cognitive outcomes [9,12,13]. The presence of cognitive deficits post-stroke increases the likelihood of long-term conditions like dementia, particularly among older patients [9,14].

Post-stroke depression (PSD) is a common complication, occurring in about one-third of stroke patients [5], and is associated with poor functional and cognitive outcomes. Depression can develop both in the acute and chronic phases of stroke, with various factors such as stroke severity, lesion location, and pre-existing psychological conditions influencing its onset and severity. PSD negatively impacts rehabilitation by reducing motivation and cognitive recovery, leading to increased dependency [15]. Predictive factors for PSD include younger age, female sex, and pre-existing depressive symptoms, while the presence of post-stroke apathy and cognitive impairments also increases the risk [9]. Early detection and management of PSD are crucial, as they are associated with better functional recovery and improved quality of life.

Several factors have been studied as potential predictors of cognitive and emotional outcomes post-stroke. Demographic variables such as younger age and higher education are strongly associated with better cognitive recovery [16], while clinical markers like lower stroke severity (measured by NIHSS), better functional outcomes (measured by mRS and Barthel Index), and lower infarct volume on imaging also contribute to improved cognitive performance [12,13]. For emotional outcomes, factors such as female sex, history of depression, and the presence of cognitive impairments are associated with a higher risk of PSD [5,9,17]. Functional status and cognitive deficits in domains like executive function, attention, and memory significantly predict long-term outcomes and are influenced by both demographic and clinical variables [18,19]. The literature acknowledges the importance of early neuropsychological assessments in predicting recovery [20,21], but challenges remain in determining their precise predictive value, especially considering the variety of cognitive impairments and the lack of standardized approaches to diagnosis.

Previous research has developed various predictive models aimed at estimating post-stroke cognitive and emotional outcomes. Specifically, Guo et al. [22] reviewed clinical models for post-stroke depression (PSD) and provided early-stage risk assessment for PSD based on clinical markers within the first week of stroke onset. Similarly, Richter et al. [23] proposed a study protocol focused on identifying prognostic markers for PSD, emphasizing the integration of clinical and biological data to improve predictive accuracy. Additionally, other researchers [24] applied deep learning with multimodal data sources, such as neuroimaging and clinical records, to forecast post-stroke cognitive recovery, using explainable AI for enhanced feature selection. Further, a meta-analysis [25] was conducted to assess the effectiveness of machine learning models in predicting post-stroke cognitive impairment (PSCI), demonstrating significant advancements in prediction accuracy yet also highlighting ongoing limitations in model consistency across patient groups. Hbid et al. [26] developed a patient-specific model to predict long-term cognitive decline, focusing on changes over a five-year period post-stroke.

Despite these advancements, existing models often lack consistency and generalizability across diverse patient populations, largely due to differences in methodologies, predictor variables, and statistical techniques. Our study aims to address these gaps by integrating a broader set of demographic and clinical markers to create a more standardized and comprehensive predictive model for post-stroke cognitive recovery and depression. In this way, this study seeks to enhance prediction accuracy and reliability, thereby improving the model’s applicability to diverse patient populations and supporting more targeted rehabilitation approaches.

## 2. Materials and Methods

This was a longitudinal study that examined patients with IS both during the acute phase of their hospitalization and in the chronic phase, 6 months post-stroke. All data were collected in accordance with the Declaration of Helsinki and in compliance with the Scientific Council of the University General Hospital of Alexandroupolis. Written informed consent was obtained from all participants in the study. The data were analyzed anonymously.

### 2.1. Participants

Patients were included in the study if they met the following criteria: age over 18 years, radiologically confirmed first-ever IS with symptom onset within 24 h and Greek as their native or speaking language. Additionally, patients needed to have no other neurological diseases apart from stroke, no history of major psychiatric disorders (such as major depressive disorder, schizophrenia, or bipolar disorder), and no history of alcohol or drug abuse. IS had to be confirmed by MRI, and the stroke needed to have occurred between 5 and 7 days prior to the assessment.

Participants were excluded if they had severe motor or sensory deficits (such as visual or hearing impairments) that could hinder cognitive performance, severe aphasia or dysarthria that would interfere with completing the study assessments, expressive or receptive aphasia at the time of examination, or an altered state of consciousness. Additionally, patients with cognitive impairment or impaired activities of daily living (ADL) reported by informants prior to the stroke, those with primary hemorrhagic stroke, acute neurological illnesses other than stroke, or a premorbid Axis I psychiatric disorder were also excluded from the study.

### 2.2. Demographic and Clinical Markers

Demographic details, such as age, sex, and educational background, were gathered from all participants. Clinical information recorded for each individual encompassed the duration of the disease (number of days since symptom onset), stroke type (only IS was recorded), and the hemisphere affected (right, left, or both). All patients underwent brain MRI on a 3.0 T MR scanner and neuroimaging data were evaluated by an experienced neuroradiologist (E.P.).

The assessment of clinical markers was conducted upon the patient’s admission to the hospital. The clinical severity of stroke was assessed using the National Institutes of Health Stroke Scale (NIHSS) [27]. This scale serves as a critical tool for healthcare professionals to measure the extent of neurological impairment in stroke patients. It consists of 15 different items that evaluate a range of functions, such as consciousness, motor abilities, coordination, and language. The total score can range from 0 to 42, with higher scores reflecting more severe strokes. The NIHSS plays a crucial role in guiding clinical decisions, determining the appropriate level of care, and monitoring changes in the patient’s condition over time. However, it is just one aspect of the comprehensive evaluation required for stroke survivors.

In addition to the NIHSS, the functional disability of stroke patients was measured using the Modified Rankin Scale (mRS) [28] and the Barthel Index (BI) [29]. The mRS is a widely recognized tool in neurology that simplifies the assessment of functional impairment, grading patients on a scale from 0 (no symptoms) to 6 (death). This tool enables clinicians to efficiently classify a patient’s level of disability, making it easier to guide treatment plans and track progress over time. The mRS is particularly useful in both acute and long-term care management, helping determine the patient’s recovery trajectory.

The Barthel Index (BI), on the other hand, evaluates the patient’s ability to perform essential activities of daily living, such as eating, dressing, and mobility. Scoring is out of 100, with higher scores indicating a higher level of functional independence [30]. This index is instrumental for rehabilitation professionals in designing individualized treatment plans that focus on improving the patient’s quality of life and restoring autonomy after a stroke.

### 2.3. Neuropsychological Markers

A neuropsychological evaluation took place within the acute phase (i.e., 5–7 days post-stroke) and was conducted by an experienced neuropsychologist (A.T.) The following neuropsychological screening measures and tests, which are standardized in the local population, were administered:

- The Montreal Cognitive Assessment (MoCA) is one of the few tools specifically designed to detect mild cognitive impairment (MCI) [31,32]. It uses a 30-point scale to evaluate language, short-term memory, visuospatial skills, attention, working memory, executive function, language, and orientation. The MoCA emphasizes tasks related to frontal lobe executive functioning, making it potentially more sensitive than the MMSE in detecting non-Alzheimer’s dementia. Research has shown that cut-off scores between 23 and 26 provide good sensitivity and specificity for identifying MCI. Additionally, it is recommended to add one point to the total score for individuals with 12 or fewer years of education to account for educational background.
- The Addenbrooke’s Cognitive Examination III (ACE-III) [33,34] is a thorough cognitive screening tool that evaluates several cognitive functions, including memory, language, attention, and visuospatial skills. It is commonly used by healthcare providers, especially neurologists and geriatricians, to detect and track cognitive impairments. The ACE-III assesses five cognitive domains: attention and orientation, memory, verbal fluency, language, and visuospatial abilities, with tasks and questions resulting in a maximum score of 100. A higher score reflects better cognitive performance, while lower scores may indicate cognitive decline. The ACE-III also incorporates elements from the Mini-Mental State Examination (MMSE) [35,36], another widely used cognitive screening test that assesses orientation, memory, attention, language, and visuospatial skills. The MMSE score ranges from 0 to 30, with lower scores suggesting more severe cognitive impairment.
- The Trail Making Test (TMT) is one of the most commonly used neuropsychological test in clinical practice. It was originally developed to measure divided attention [37]. Part A of the TMT (TMT-A) evaluates attention, visual scanning, eye–hand coordination, and processing speed, while Part B (TMT-B) focuses more on the ability to switch between different cognitive sets [38,39]. These cognitive processes are essential for executive functioning [40,41].
- The Boston Naming Test (BNT) is a confrontation naming assessment commonly used in clinical settings to identify mild word-retrieval difficulties in individuals with aphasia, brain injuries, or dementia [42,43].
- The verbal fluency test evaluates an individual’s capacity to quickly generate words based on a given criterion, such as starting with a specific letter (e.g., H, S, or A) or belonging to a particular semantic category (e.g., animals, fruits, objects). These tests challenge several cognitive processes, and difficulties or errors during the task may indicate impairments in various areas, including attention, working memory, semantic memory, executive function, and language skills. Specifically, attention is required to stay focused on the task, working memory helps to keep track of words already said, semantic memory provides access to vocabulary and knowledge, and executive functioning is crucial for organizing thoughts and adhering to the task rules. As a result, verbal fluency test is a valuable tool in assessing cognitive flexibility, retrieval efficiency, and overall language function, often used in diagnosing or monitoring conditions like dementia, brain injury, or other neurological disorders [40,44].
- The Functional Cognitive Assessment Scale (FUCAS) was administered to assess executive cognitive function in ADL [45].
- The Hamilton Depression Scale (HAM-D) was used to evaluate the current emotional state [46].

### 2.4. Statistical Analysis

Demographic characteristics (age, sex, education) and clinical data (stroke laterality, NIHSS, mRS, BI) were used as predictors. Dependent variables were the following cognitive measures: MMSE, MoCA, ACE-III, TMT-A, TMT-B, BNT, Fluency Semantic, Fluency Phonemic, and FUCAS. The Generalized Linear Mixed Model (GLMM) analysis was applied ten times, once for each dependent variable. In all ten analyses, the same seven independent variables were used. The seventeen variables are shown in Table 1, along with their descriptive statistics. For the dependent variables MMSE, MoCA, ACE, and HAMILTON, which are categorical variables with two categories, the Binary Logistic model type was used. For the variables TMT-A, TMT-B, BNT, Fluency Semantic, Fluency Phonemic, FUCAS, and HAM-D, the Gamma model with a log link was used. The last choice was made because these variables did not follow either the normal distribution or the Poisson distribution, and the data exhibited overdispersion. The Gamma model with a log link was preferred over the Negative Binomial model because, in all cases, it yielded lower values for the Akaike Corrected (AICc) and Bayesian Information Criterion (BIC), indicating better model fit to the data. All statistical analyses were performed using the SPSS statistical package (version 25.00), with a significance level set at *p* < 0.05.

Our analysis shows higher predictive accuracy compared to models by other researchers, who either reported lower accuracy rates or did not provide specific percentages for comparison. One of the key strengths of our approach is the use of models, which allows us to assess the simultaneous impact of multiple factors on the dependent variable. In these models, we can identify which factors genuinely influence the dependent variable by accounting for other variables simultaneously. This leads to a more accurate understanding of the true relationship between the dependent variable and the factors in question. In contrast, univariate approaches such as **t**-Tests, ANOVA, or simple correlations evaluate each factor in isolation, which can result in oversimplified or misleading conclusions [47]. Furthermore, by analyzing multiple variables at once, Mixed Models approaches reduce the risk of multiple comparisons and the increased likelihood of a Type I error, which often arises when performing numerous univariate tests. This is a common issue in public health research, where Type I error rates frequently exceed the traditional 5% threshold [48].

## 3. Results

Table 1 presents the descriptive statistics for all the variables used in the ten analyses (distinguishing between independent and dependent variables). The variables are shown in the form (categorical or continuous) in which they were used in the statistical analyses and are presented separately for the time points 0 and 1. For categorical variables, the frequency is shown, while for continuous variables, the mean (±standard deviation) is presented.

Table 2 presents the results for the four categorical dependent variables. For the MMSE, the probability of “no impairment” increases as education increases (*p* < 0.001). Similarly, for the MoCA, the probability of “no impairment” increases with higher education (*p* = 0.002). For the ACE-III, the probability of “no impairment” increases with decreasing age (*p* = 0.017), higher education (*p* = 0.001), and decreasing mRS (*p* = 0.012). For the HAM-D, the probability of being without depression increases in males, and as the education increases (*p* = 0.01) and BI score increases (*p* < 0.001). The accuracy of the models (percentage of successful predictions) ranges from 87.7% to 95.7%.

Table 3 presents the results for the six continuous dependent variables. TMT-A increases in females (*p* = 0.009), as well as with increasing age (*p* < 0.001), higher education (*p* < 0.001), increasing mRS (*p* < 0.001), and decreasing BI (*p* = 0.034). TMT-B increases with increasing age (*p* < 0.001), higher education (*p* < 0.001), decreasing NIHSS (*p* = 0.005), and increasing mRS (*p* < 0.001). BNT increases with higher education (*p* < 0.001). FUCAS increases with increasing age (*p* = 0.008) and higher education (*p* = 0.001). Negative coefficients were observed for education in relation to TMT-A and FUCAS scores, indicating that higher educational levels were associated with reduced completion times in TMT-A and lower FUCAS scores. This suggests that individuals with higher education may exhibit better processing speed (TMT-A) and functional outcomes (FUCAS), potentially due to increased cognitive reserve and better coping mechanisms. Fluency Semantic increases with decreasing age (*p* = 0.014) and higher education (*p* = 0.003) and Fluency Phonemic increases with higher education (*p* < 0.001). The performance of the models (correlation between actual and predicted values) ranges from 88.9% to 98%.

### Model Accuracy

The results of the statistical analysis demonstrated high model accuracy across both categorical and continuous dependent variables, indicating that the models were well-fitted to the data. For the categorical variables, the models achieved a high level of accuracy, ranging from 87.7% to 95.7%. Specifically, the predictor variables, including education, age, and mRS scores, were significant factors influencing cognitive impairment outcomes. Specifically, the results indicated the importance of educational attainment in cognitive functioning. Similarly, the Gamma models used for the continuous dependent variables demonstrated robust accuracy, with the models’ performance ranging from 88.9% to 98%, suggesting that the selected predictor variables, such as age, education, and mRS, were highly predictive of these outcomes. Overall, the high accuracy rates indicate that the models used in this study provided reliable predictions of both categorical and continuous outcomes. The consistent impact of key variables like education, age, and mRS across different cognitive measures reinforces their role as important determinants of cognitive performance.

## 4. Discussion

### 4.1. Overall Value and Clinical Utility of the Predictive Model

Ιn this study, a longitudinal analysis using GLMM was conducted to identify predictive markers of cognitive recovery and post-stroke depression in IS patients. We identified both demographic and clinical markers that predict cognitive outcomes and post-stroke depression six months following IS. The present study introduces a predictive model for cognitive and emotional outcomes in ischemic stroke (IS) patients that integrates a variety of demographic, clinical, and functional predictors. This model surpasses the limitations of univariate or simpler multivariate models, which often assess individual risk factors in isolation. By using a comprehensive approach that combines key predictive markers—such as age, education, stroke severity, and functional status—our model achieves high accuracy (87.7% to 98%) across both categorical and continuous outcomes. This level of accuracy indicates that the model is well-suited for application in clinical settings, offering healthcare providers a reliable tool to predict long-term recovery trajectories.

### 4.2. Influence of Key Predictive Markers Within the Model

While the model’s strength lies in its integration of multiple factors, individual predictors provide essential contributions. Here, we briefly summarize the roles of demographic and clinical markers, highlighting how each variable contributes to the model’s overall predictive power.

#### 4.2.1. Demographic Markers

Demographic factors, such as age, education, and sex, are key predictors of post-stroke outcomes and significantly contribute to the model’s predictive strength by capturing individual differences in baseline resilience and cognitive reserve.

Age emerged as a significant factor in various outcomes. For the categorical outcome ACE-III, younger age was associated with a higher probability of no impairment. Previous research [49] compares the ACE and MoCA tests, noting that both are useful for detecting cognitive impairments, with the ACE showing strong sensitivity for amnestic impairments. This sensitivity could support our finding, as younger individuals, who typically have better memory and faster processing speeds, may have a higher likelihood of showing no impairments. However, the study does not specifically address age as a factor. Similarly, Blackburn [19] highlights the sensitivity of the ACE in detecting cognitive impairment in post-stroke patients, suggesting that many impairments are missed by other tests, like the MMSE. However, these studies do not focus on age, but the overall findings align with our research, suggesting that younger individuals may have fewer cognitive impairments detectable by the ACE.

In continuous outcomes, older age was linked with worse performance (increased time) on both TMT-A and TMT-B, indicating slower processing speed and impaired mental flexibility/executive function. These findings are in accordance with previous research [50], which found that in a sample of 70-year-old men, cognitive function as measured by TMT-B was a strong predictor of future brain infarction, independently of other factors such as education and social status. Shao [18] also observed significantly longer completion times and more errors on both TMT-A and TMT-B among mild stroke patients compared to controls. The correlation between TMT performance and global cognition highlights the sensitivity of these tasks in detecting cognitive decline, particularly executive dysfunction. The findings strongly parallel the effects of aging, further supporting the link between older age and reduced cognitive performance on these tasks.

Age also negatively influenced Fluency Semantic, suggesting reduced language-related cognitive function as age increases. This finding is well-supported in the literature. Verbal fluency tasks, which measure both verbal ability and executive function, rely on various cognitive processes, including attention and cognitive flexibility, functions that decline with age due to the deterioration of frontal brain regions [51]. This aligns with our finding, as the loss of executive control over word retrieval can explain reduced fluency in older individuals. Brady [52] further supports that age is consistently associated with a decline in cognitive functions, particularly verbal fluency, which is also affected by stroke risk factors. Brady’s findings further indicate that the decline in fluency due to aging mirrors similar declines in memory and visuospatial performance, pointing to a broad cognitive deterioration linked to age. Together, these studies support the idea that aging leads to reduced fluency and language-related cognitive functions.

Sex influenced several outcomes, with notable effects in HAM-D and TMT-A. For the HAM-D scale, males were more likely to be categorized as “normal” compared to females. This finding aligns with evidence from several studies examining sex differences in PSD. Volz [53] et al. found that women had higher PSD prevalence and severity shortly after stroke, though these differences diminished over time, disappearing within six months. Dong et al. [54] similarly noted that women were more likely to have a history of depression and be on medication for depression at the time of stroke, but no significant sex differences in PSD were observed 90 days post-stroke after adjusting for sociodemographic factors. Mayman et al. [55] further supported this by showing that women had a 20% higher risk of developing PSD than men, with this risk persisting even 1.5 years post-stroke. These studies suggest that women exhibit more severe or prolonged depressive symptoms post-stroke, which may explain their lower likelihood of being categorized as “normal” on the HAM-D scale compared to men.

For TMT-A, females had significantly longer completion times than males, indicating slower processing speeds among females. This finding is supported by some existing literature while remaining underexplored in other studies. Roivainen et al. [56] reviewed sex differences in processing speed and found that while females perform faster on language-related tasks, males tend to perform better on tasks requiring motor speed and reaction time, such as TMT-A, aligning with our observation of slower completion times for females. Shao et al. [18] further support the sensitivity of TMT-A in detecting slower processing speeds, particularly in stroke patients, though the study does not specifically address sex differences. Shao’s research emphasized that both processing speed (TMT-A) and cognitive flexibility (TMT-B) were impaired in stroke patients, reinforcing the idea that slower cognitive and motor processing speeds can be captured by these tasks. However, Wiberg et al. [50] primarily focused on cognitive function and its link to stroke risk without addressing sex differences, leaving a gap in the exploration of sex-specific outcomes in TMT performance.

Education was a consistently strong predictor across multiple outcomes. Higher levels of education were associated with a greater likelihood of no impairment in MMSE, MoCA, and ACE-III. This result is supported by several studies. Pendlebury et al. [49] highlighted that both the MoCA and ACE were sensitive tools for measuring cognitive outcomes, particularly in amnestic impairments, and that these tools can be useful for patients with varying educational backgrounds, given that educated individuals may possess stronger cognitive reserve that enhances their ability to perform well on such assessments. Another study [57] further supports this finding, noting that lower education was independently associated with non-feasibility of MoCA testing in acute stroke patients, indicating that individuals with higher education were more likely to complete the MoCA without cognitive impairment being detected. Researchers [4] also emphasized that MoCA is more sensitive than the MMSE for detecting cognitive impairment post-stroke, and education was identified as a key factor influencing cognitive test performance, supporting the link between higher education and better outcomes on these cognitive screening tools. Similarly, Pasi et al. [58] found that low education levels were a significant predictor of poorer MoCA performance, reinforcing the idea that higher educational attainment improves cognitive outcomes post-stroke. Ιn contrast, Blackburn et al. [19] highlighted the ACE sensitivity in detecting cognitive impairments in post-stroke settings but did not address the role of education. Cova et al. [59] focused on age and cognitive deficits as predictors of cognitive decline, while other researchers [11,50] emphasized cognitive recovery without exploring the impact of education.

Education also influenced performance on BNT, Fluency Semantic, and Fluency Phonemic, highlighting the role of education in preserving language and executive cognitive processes after stroke. This observation highlights the role of education in preserving cognitive function after stroke and is supported by several studies. Fishman et al. [51] noted that verbal fluency tasks are influenced by vocabulary knowledge, which is highly associated with level of education [60]. Additionally, Brady at al. [52] found that although age and stroke risk contribute to declines in verbal fluency, education was a protective factor, mitigating some of the effects of stroke and aging on cognitive function. Higher education was associated with better outcomes in fluency tasks, emphasizing its role in maintaining cognitive function after stroke.

Finally, lower education was associated with worse performance on both TMT-A and TMT-B. Previous research [38,61] showed that performance on TMT, especially part B, was significantly influenced by education, with lower education levels being associated with worse outcomes in both healthy individuals and stroke patients. This reinforces our finding that lower education contributes to worse TMT performance. Shao et al. [18] also found that completion times for both TMT-A and TMT-B were significantly longer in stroke patients compared to controls, reflecting impairments in processing speed and cognitive flexibility. Although Shao et al. did not specifically focus on education, the correlation between TMT performance and global cognition suggests that individuals with lower cognitive reserve, potentially linked to lower education, may struggle more with these tasks. On the other hand, Wiberg et al. [50] examined cognitive function as measured by TMT-B and its predictive value for brain infarction but did not detect an influence of education on the results. The inverse relationship between education and performance times on TMT-A, as well as lower FUCAS scores, underscores the role of educational attainment as a factor that may enhance cognitive flexibility and functional adaptation post-stroke. These results support the hypothesis that individuals with higher education can draw on a greater cognitive reserve, facilitating more efficient cognitive processing and resilience in recovery contexts [19,62]. The study emphasized the role of subcortico-frontal activities in predicting brain infarction, independent of education or social factors, suggesting that other variables might play a more prominent role in TMT performance in their cohort.

#### 4.2.2. Clinical Markers

Clinical markers of stroke severity and functional status—such as NIHSS, mRS, BI, and stroke laterality—play a vital role in predicting both cognitive and emotional recovery. These factors enable the model to assess the broader impact of the stroke on a patient’s recovery potential and functional independence.

The NIHSS score, a measure of stroke severity, was a significant predictor for TMT-B, with lower NIHSS scores predicting better performance. However, NIHSS did not significantly predict outcomes for other neuropsychological testing. The connection between lower NIHSS scores and better performance on TMT is well-supported. Research has shown that both TMT-A and TMT-B are significantly correlated with stroke severity, with lower NIHSS scores linked to better performance, suggesting that less severe strokes allow for better cognitive flexibility [63]. Similarly, baseline NIHSS scores have been identified as strong predictors of stroke outcomes, where lower scores correlate with better cognitive recovery, further supporting the relationship between stroke severity and TMT performance [64]. Impairments in processing speed and cognitive flexibility are commonly observed in stroke patients, indicating that more severe strokes would impair these abilities, even though not all studies directly examine NIHSS scores in this context [18].

The mRS score, which measures the degree of disability or dependence after stroke, was significantly related to multiple outcomes. A lower mRS score, indicating better functional recovery, was associated with no impairment in ACE-III. Higher mRS scores were associated with worse performance on TMT-A and TMT-B, reflecting the influence of functional disability on cognitive processing and executive function. Previous research has shown that lower mRS scores are linked with better cognitive performance and functional independence, reinforcing the connection between functional recovery and cognitive outcomes [65]. The sensitivity of the ACE in detecting cognitive impairments, particularly in the non-acute post-stroke setting, suggests that better functional recovery is likely to lead to better performance on ACE [49]. Furthermore, longer completion times on TMT-A and TMT-B have been shown to reflect impairments in processing speed and cognitive flexibility in stroke patients, which aligns with the finding that higher mRS scores, indicating worse functional recovery, lead to worse performance on these tasks [18]. Although some studies, such as those by Wiberg [50], did not explore functional recovery, the overall evidence supports the idea that lower mRS scores contribute to better cognitive outcomes on tests like ACE and TMT-A and TMT-B.

The BI, which assesses the patient’s ability to perform activities of daily living, was a significant predictor for HAM-D, with higher BI scores associated with normal scores on the depression scale. It also had a marginal impact on TMT-A, suggesting a link between physical and cognitive recovery. Research has shown that functional recovery, as measured by the BI, is a strong predictor of both physical and cognitive recovery in stroke patients. Higher BI scores are linked to better cognitive outcomes and greater independence in ADL [66,67], which aligns with our findings that higher BI scores predict better HAM-D outcomes. Similarly, it has been found that physical recovery, as reflected in higher BI scores, can also influence cognitive performance, such as faster completion times on TMT-A, which measures processing speed [68]. This suggests that physical and cognitive recovery are interconnected, supporting our observation of the link between BI scores and cognitive outcomes. While other researchers [46,52] focus more on PSD and cognitive impairment, they emphasize how stroke severity and functional deficits predict poorer outcomes, indirectly supporting the idea that better functional recovery is associated with improved cognitive and emotional outcomes in stroke patients [69,70].

Stroke laterality did not significantly influence the examined outcomes. Previous studies found no significant differences in cognitive outcomes, such as MMSE scores, between patients with left or right hemisphere lesions, suggesting that the affected hemisphere may not play a critical role in overall cognitive recovery [71]. Similarly, a study examining hemispheric lateralization in stroke outcomes found no significant difference between right and left hemisphere stroke patients in terms of 90-day functional outcomes, including modified Rankin Scale (mRS) scores and mortality [72]. The HERMES meta-analysis also concluded that stroke lateralization did not significantly modify outcomes, further reinforcing the idea that the hemisphere affected by the stroke does not strongly influence recovery outcomes [73]. More detailed evaluations of brain structural and functional networks (e.g., using neuroimaging techniques such as diffusion tensor imaging and functional magnetic resonance imaging) may be necessary to further evaluate the predictive of baseline brain integrity with regard to cognitive and emotional outcome.

### 4.3. Strengths and Limitations

The study presents several strengths and limitations. One of its key strengths is the longitudinal design, which allows for the tracking of recovery over time, providing a clearer understanding of both short-term and long-term outcomes. Additionally, the use of comprehensive neuropsychological assessments ensures that a wide range of cognitive domains, such as memory, attention, and executive function, are thoroughly evaluated. The inclusion of both cognitive screening measures, individual cognitive tests, and functional cognitive assessment scales, as well as emotional inventories, adds depth to the analysis by addressing not only cognitive but also emotional recovery. Furthermore, the high model accuracy (ranging from 87.7% to 98%) highlights the reliability of the chosen predictors, such as age, education, and the modified Rankin Scale (mRS), in forecasting recovery outcomes. By leveraging multivariate analysis, our study offers a more robust and accurate model for predicting outcomes, reducing error rates, and providing a comprehensive understanding of the key predictive factors.

However, the study also has some limitations. The sample size and population are confined to Greek-speaking patients with IS, which may reduce the generalizability of the results to other populations, including patients with hemorrhagic stroke, which was not included in the present study. In addition, the exclusion of patients with severe aphasia or other neurological conditions limits the applicability of the findings to a broader stroke population, particularly those with more severe impairments. Furthermore, considering the acute phase of the IS during which the neuropsychological evaluation took place, an individual test of verbal/visual learning/memory was not administered to minimize patient’s fatigue. The limited follow-up duration is another limitation, as more long-term follow-up evaluations could provide valuable insights into the sustainability of the predictive model over time. Another limitation of our study is the lack of assessment of bilingualism among participants. Bilingualism can influence cognitive recovery in stroke patients, especially in language processing and executive function. Since we did not specifically evaluate or control for bilingualism, this may introduce variability in our cognitive recovery findings. Future studies should consider including bilingualism as a variable. Additionally, we did not collect data on handedness or brain lateralization at baseline or follow-up. These factors can impact cognitive and motor recovery depending on stroke hemisphere. Future research incorporating these variables could provide deeper insights into their role in post-stroke recovery. Finally, while the predictive accuracy of our models was high within this sample, the homogeneity of the Greek-speaking ischemic stroke population may have contributed to this outcome. Applying the model to more diverse and heterogeneous samples could further validate its predictive value and offer insights into factors that may influence accuracy across varied populations. Future studies should aim to replicate and test these models in broader cohorts, including patients with different stroke types and backgrounds, to confirm the model’s generalizability and robustness.

### 4.4. Clinical Implications and Further Research

The results of the present study can be valuable not only for the diagnostic process of patients with IS but also for their ongoing monitoring and the adaptation of appropriate therapeutic interventions throughout the rehabilitation phase. The use of specific neuropsychological tests with predictive value can significantly contribute to a multidisciplinary approach to IS care, offering essential data for tracking recovery progress. Such assessments can inform healthcare teams about post-stroke cognitive and emotional statuses early on, allowing for tailored rehabilitation strategies that align with individual patient needs. Moreover, rehabilitation centers can integrate these findings into their protocols, enhancing the effectiveness of treatment by providing structured, evidence-based data that emphasize the importance of interdisciplinary collaboration. By incorporating neuropsychological evaluations with proven prognostic value, healthcare professionals can more accurately predict recovery outcomes and adjust their interventions accordingly, ultimately improving patient care and long-term functional independence. This holistic approach, which combines neuropsychological assessment and interdisciplinary efforts, reinforces the role of collaboration between neurologists, neuropsychologists, psychologists, physiotherapists, and other healthcare professionals in optimizing stroke rehabilitation outcomes.

Further research should focus on validating these findings across more diverse populations and extending follow-up periods to better assess long-term recovery patterns and refine the predictive models for broader clinical applicability. This will help in developing more standardized neuropsychological assessments and rehabilitation approaches tailored to individual patient profiles.

## 5. Conclusions

This study identified education and age as the strongest predictors of cognitive recovery in IS patients, with higher education levels consistently linked to better outcomes, while functional status played a critical role in predicting both cognitive performance and emotional well-being (i.e., PSD). The predictive models used in the analysis demonstrated high accuracy, with performance rates ranging from 87.7% to 98%, underscoring the reliability of these variables in forecasting recovery outcomes. These findings emphasize the importance of considering patients’ demographic profile and integrating functional, cognitive and emotional rehabilitation strategies to optimize post-stroke recovery, and also suggest the need for further studies with diverse populations and longer follow-up durations to enhance the generalizability and long-term applicability of the predictive models.

## Figures and Tables

**Table 1:** Descriptive statistics for independent and dependent variables at time points 0 and 1.

Variables			TIME	
			t0	t1
Independent	Age (yrs)		63.01 ± 11.89	
	Education (yrs)		8.36 ± 5.13	
	Sex	M (n)	45	
		F (n)	24	
	Hemisphere	L (n)	33	
		R (n)	36	
	NIHSS		4.67 ± 3.85	1.17 ± 1.43
	mRS		2.45 ± 1.5	1.01 ± 1.08
	BI		86.45 ± 21.34	92.83 ± 14.05
Dependent	MMSE	Imparment (n)	21	20
		No impairment (n)	48	49
	MoCA	Imparment (n)	53	49
		No impairment (n)	16	20
	ACE-III	Imparment (n)	60	53
		No impairment (n)	9	16
	HAM-D	Depression	25	26
		No depression (n)	44	43
	TMT-A (s)		232.52 ± 206.56	169.25 ± 176.56
	TMT-B (s)		337.87 ± 191.13	303.38 ± 195.06
	BNT		37.26 ± 12.95	38.49 ± 13.05
	Fluency semantic		29.22 ± 13.84	31.96 ± 13.39
	Fluency phonemic		18.62 ± 12.54	19.72 ± 12.99
	FUCAS		48.57 ± 15.78	47.68 ± 14.21

Notes. t0 = time 0 (baseline, acute phase); t1 = time 1 (follow-up, 6 months post-stroke); yrs = years; n = number of patients; M = male; F = female; L = left; R = right; NIHSS = National Institutes of Health Stroke Scale; mRS = modified Rankin Scale; BI = Barthel Index; MMSE = Mini-Mental State Examination; MoCA = Montreal Cognitive Assessment; ACE-III = Addenbrooke’s Cognitive Examination-III; HAM-D = Hamilton Depression Scale; TMT-A = Trail Making Test-part A; TMT-B = Trail Making Test-part B; s = seconds; BNT = Boston Naming Test; FUCAS = Functional Cognitive Assessment Scale.

**Table 2:** Summary of model coefficients (b), *p*-values and models’ accuracy for the categorical dependent variables.

Dependent Variable	MMSE		MoCA		ACE-III		HAM-D	
Reference category	Impairment		Impairment		Impairment		Depression	
Model	No impairment		No impairment		No impairment		No depression	
Model Term	b	*p*	b	*p*	b	*p*	b	*p*
Age	0.083	0.521	−0.278	0.086	−0.290	**0.017**	0.093	0.386
Sex = 1 (M)	4.220	0.220	1.121	0.748	−2.674	0.303	9.917	**0.000**
Sex = 2 (F)	0.000	N/A	0.000	N/A	0.000	N/A	0.000	N/A
Education	1.639	**0.000**	1.265	**0.002**	0.912	**0.001**	0.624	**0.010**
NIHSS t0	−0.738	0.620	−0.323	0.565	0.378	0.270	1.080	0.336
mRS t0	−1.743	0.597	−2.961	0.164	−3.416	**0.012**	3.288	0.108
BI t0	0.232	0.281	0.025	0.828	−0.027	0.701	0.604	**0.000**
Hem = 1 (L)	−1.377	0.690	1.156	0.735	−0.476	0.838	−1.050	0.674
Hem = 2 (R)	0.000	N/A	0.000	N/A	0.000	N/A	0.000	N/A
Model’s accuracy	94.2%		95.7%		94.9%		87.7%	

Notes. M = male; F = female; L = left; R = right; NIHSS = National Institutes of Health Stroke Scale; mRS = modified Rankin Scale; BI = Barthel Index; t0 = time 0 (baseline, acute phase); MMSE = Mini-Mental State Examination; MoCA = Montreal Cognitive Assessment; ACE-III = Addenbrooke’s Cognitive Examination-III; HAM-D = Hamilton Depression Scale; N/A: not applicable. Bold *p*-values correspond to significant predictors at *p* < 0.05.

**Table 3:** Summary of model coefficients (b), *p*-values, and models’ accuracy for the continuous dependent variables.

Dependent Variable	TMT-A		TMT-B		BNT		Fluency Semantic		Fluency Phonemic		FUCAS	
Model Term	b	*p*	b	*p*	b	*p*	b	*p*	b	*p*	B	*p*
Age	0.019	**0.000**	0.019	**0.000**	−0.004	0.188	−0.009	**0.014**	−0.005	0.317	0.005	**0.008**
Sex = 1 (M)	−0.415	**0.009**	−0.074	0.558	0.074	0.248	−0.013	0.908	−0.058	0.686	−0.041	0.391
Sex = 2 (F)	0.000	N/A	0.000	N/A	0.000	N/A	0.000	N/A	0.000	N/A	0.000	N/A
Education	−0.071	**0.000**	−0.083	**0.000**	0.036	**0.000**	0.033	**0.003**	0.067	**0.000**	−0.007	**0.001**
NIHSS t0	−0.043	0.050	−0.029	**0.005**	−0.003	0.577	0.005	0.582	0.001	0.925	−0.002	0.470
mRS t0	0.272	**0.000**	0.153	**0.000**	−0.017	0.356	−0.051	0.084	−0.050	0.096	0.013	0.077
BI t0	−0.011	**0.034**	−0.004	0.277	−0.000	0.837	0.003	0.237	0.003	0.329	−0.001	0.268
Hem = 1 (L)	0.082	0.547	−0.040	0.731	0.002	0.977	−0.107	0.265	−0.030	0.834	0.069	0.128
Hem = 2 (R)	0.000	N/A	0.000	N/A	0.000	N/A	0.000	N/A	0.000	N/A	0.000	N/A
Model’s accuracy	94%		91.4%		98%		88.9%		98%		88.9%	

Notes. M = male; F = female; L = left; R = right; NIHSS = National Institutes of Health Stroke Scale; mRS = modified Rankin Scale; BI = Barthel Index; TMT-A = Trail Making Test-part A; TMT-B = Trail Making Test-part B; BNT = Boston Naming Test; FUCAS = Functional Cognitive Assessment Scale; N/A: not applicable. Bold *p*-values correspond to significant predictors at *p* < 0.05.

## Data Availability

Data available upon reasonable request.

## References

[1] Rothenburg L.S., Herrmann N., Swardfager W., Black S.E., Tennen G., Kiss A., Gladstone D.J., Ween J., Snaiderman A., Lanctôt K.L. (2010). The Relationship between Inflammatory Markers and Post Stroke Cognitive Impairment. J. Geriatr. Psychiatry Neurol..

[2] Sagnier S., Munsch F., Bigourdan A., Debruxelles S., Poli M., Renou P., Olindo S., Rouanet F., Dousset V., Tourdias T. (2019). The Influence of Stroke Location on Cognitive and Mood Impairment. A Voxel-Based Lesion-Symptom Mapping Study. J. Stroke Cerebrovasc. Dis..

[3] Barker-Collo S., Starkey N., Lawes C.M.M., Feigin V., Senior H., Parag V. (2012). Neuropsychological Profiles of 5-Year Ischemic Stroke Survivors by Oxfordshire Stroke Classification and Hemisphere of Lesion. Stroke.

[4] Washida K., Ihara M., Tachibana H., Sekiguchi K., Kowa H., Kanda F., Toda T. (2014). Association of the ASCO Classification with the Executive Function Subscores of the Montreal Cognitive Assessment in Patients with Postischemic Stroke. J. Stroke Cerebrovasc. Dis..

[5] Tziaka E., Christidi F., Tsiptsios D., Sousanidou A., Karatzetzou S., Tsiakiri A., Doskas T.K., Tsamakis K., Retzepis N., Konstantinidis C. (2023). Leukoaraiosis as a Predictor of Depression and Cognitive Impairment among Stroke Survivors: A Systematic Review. Neurol. Int..

[6] Khan M., Heiser H., Bernicchi N., Packard L., Parker J.L., Edwardson M.A., Silver B., Elisevich K.V., Henninger N. (2019). Leukoaraiosis Predicts Short-Term Cognitive But Not Motor Recovery in Ischemic Stroke Patients During Rehabilitation. J. Stroke Cerebrovasc. Dis..

[7] Georgakis M.K., Fang R., Düring M., Wollenweber F.A., Bode F.J., Stösser S., Kindlein C., Hermann P., Liman T.G., Nolte C.H. (2023). Cerebral Small Vessel Disease Burden and Cognitive and Functional Outcomes after Stroke: A Multicenter Prospective Cohort Study. Alzheimers Dement..

[8] Jaillard A., Naegele B., Trabucco-Miguel S., LeBas J.F., Hommel M. (2009). Hidden Dysfunctioning in Subacute Stroke. Stroke.

[9] De Reuck J., De Clerck M., Van Maele G. (2006). Vascular Cognitive Impairment in Patients with Late-Onset Seizures after an Ischemic Stroke. Clin. Neurol. Neurosurg..

[10] Aben H.P., De Munter L., Reijmer Y.D., Spikman J.M., Visser-Meily J.M.A., Biessels G.J., De Kort P.L.M., PROCRAS Study Group (2021). Prediction of Cognitive Recovery After Stroke: The Value of Diffusion-Weighted Imaging-Based Measures of Brain Connectivity. Stroke.

[11] Yang Y., Ye L., Lin R., Lin H., Tang T., Chen Y., Zhang J., Wang L. (2020). Neuropsychological and Neuroimaging Assessments of Early Cognitive Impairment in Patients after Mild Ischemic Stroke and Transient Ischemic Attack. J. Integr. Neurosci..

[12] Weaver N.A., Kuijf H.J., Aben H.P., Abrigo J., Bae H.-J., Barbay M., Best J.G., Bordet R., Chappell F.M., Chen C.P.L.H. (2021). Strategic Infarct Locations for Post-Stroke Cognitive Impairment: A Pooled Analysis of Individual Patient Data from 12 Acute Ischaemic Stroke Cohorts. Lancet Neurol..

[13] Tsiakiri A., Giantsios C., Vlotinou P., Nikolaidou A., Atanbori J., Sohani B., Aliyu A., Mournou A., Peristeri E., Frantzidis C. (2024). Mapping Brain Networks and Cognitive Functioning after Stroke: A Systematic Review. Brain Organoid Syst. Neurosci. J..

[14] Mandzia J.L., Smith E.E., Horton M., Hanly P., Barber P.A., Godzwon C., Donaldson E., Asdaghi N., Patel S., Coutts S.B. (2016). Imaging and Baseline Predictors of Cognitive Performance in Minor Ischemic Stroke and Patients With Transient Ischemic Attack at 90 Days. Stroke.

[15] Lamb M., Buchanan D., Godfrey C.M., Harrison M.B., Oakley P. (2008). The Psychosocial Spiritual Experience of Elderly Individuals Recovering from Stroke: A Systematic Review. Int. J. Evid.-Based Healthc..

[16] Schellekens M.M.I., Springer R.C.S., Boot E.M., Verhoeven J.I., Ekker M.S., van Alebeek M.E., Brouwers P.J.A.M., Arntz R.M., van Dijk G.W., Gons R.A.R. (2024). Cognitive Trajectory in the First Year after First-Ever Ischaemic Stroke in Young Adults: The ODYSSEY Study. J. Neurol. Neurosurg. Psychiatry.

[17] WHO (2023). Package of Interventions for Rehabilitation. Module 3. Neurological Conditions.

[18] Shao K., Wang W., Guo S.-Z., Dong F.-M., Yang Y.-M., Zhao Z.-M., Jia Y.-L., Wang J.-H. (2020). Assessing Executive Function Following the Early Stage of Mild Ischemic Stroke with Three Brief Screening Tests. J. Stroke Cerebrovasc. Dis..

[19] Blackburn D.J., Bafadhel L., Randall M., Harkness K.A. (2013). Cognitive Screening in the Acute Stroke Setting. Age Ageing.

[20] Cumming T.B., Marshall R.S., Lazar R.M. (2013). Stroke, Cognitive Deficits, and Rehabilitation: Still an Incomplete Picture. Int. J. Stroke.

[21] Pasotti F., Magnani F.G., Gallucci M., Salvato G., Ovadia D., Scotto M., Merolla S., Beretta S., Micieli G.R., Agostoni E.C. (2020). Neuropsychological Assessment in Acute Stroke Patients. Neurol. Sci..

[22] Guo J., Wang J., Sun W., Liu X. (2022). The Advances of Post-Stroke Depression: 2021 Update. J. Neurol..

[23] Richter D., Ebert A., Mazul-Wach L., Ruland Q., Charles-James J., Gold R., Tsivgoulis G., Juckel G., Krogias C. (2022). Prognostic Markers of Post-Stroke Depression (PROMoSD): Study Protocol of a Prospective Single-Center Observational Study on Raphe Hypoechogenicity as a Predictor of Post-Stroke Depression. Neurol. Res. Pract..

[24] White A., Saranti M., Garcez A.D.A., Hope T.M.H., Price C.J., Bowman H. (2024). Predicting Recovery Following Stroke: Deep Learning, Multimodal Data and Feature Selection Using Explainable AI. NeuroImage Clin..

[25] Li X., Chen Z., Jiao H., Wang B., Yin H., Chen L., Shi H., Yin Y., Qin D. (2023). Machine Learning in the Prediction of Post-Stroke Cognitive Impairment: A Systematic Review and Meta-Analysis. Front. Neurol..

[26] Hbid Y., Fahey M., Wolfe C.D.A., Obaid M., Douiri A. (2021). Risk Prediction of Cognitive Decline after Stroke. J. Stroke Cerebrovasc. Dis..

[27] Brott T., Adams H.P., Olinger C.P., Marler J.R., Barsan W.G., Biller J., Spilker J., Holleran R., Eberle R., Hertzberg V. (1989). Measurements of Acute Cerebral Infarction: A Clinical Examination Scale. Stroke.

[28] Kasner S.E. (2006). Clinical Interpretation and Use of Stroke Scales. Lancet Neurol..

[29] Collin C., Wade D.T., Davies S., Horne V. (1988). The Barthel ADL Index: A Reliability Study. Int. Disabil. Stud..

[30] McDowell I., Newell C. (1996). Measuring Health: A Guide to Rating Scales and Questionnaires.

[31] Nasreddine Z.S., Phillips N.A., Bédirian V., Charbonneau S., Whitehead V., Collin I., Cummings J.L., Chertkow H. (2005). The Montreal Cognitive Assessment, MoCA: A Brief Screening Tool for Mild Cognitive Impairment. J. Am. Geriatr. Soc..

[32] Tsiakiri A., Vadikolias K., Tripsianis G., Vlotinou P., Serdari A., Terzoudi A., Heliopoulos I. (2021). Influence of Social and Demographic Factors on the Montreal Cognitive Assessment (MoCA) Test in Rural Population of North-Eastern Greece. Geriatrics.

[33] So M., Foxe D., Kumfor F., Murray C., Hsieh S., Savage G., Ahmed R.M., Burrell J.R., Hodges J.R., Irish M. (2018). Addenbrooke’s Cognitive Examination III: Psychometric Characteristics and Relations to Functional Ability in Dementia. J. Int. Neuropsychol. Soc..

[34] Kourtesis P., Margioti E., Demenega C., Christidi F., Abrahams S. (2020). A Comparison of the Greek ACE-III, M-ACE, ACE-R, MMSE, and ECAS in the Assessment and Identification of Alzheimer’s Disease. J. Int. Neuropsychol. Soc..

[35] Folstein M.F., Folstein S.E., McHugh P.R. (1975). “Mini-Mental State”. A Practical Method for Grading the Cognitive State of Patients for the Clinician. J. Psychiatr. Res..

[36] Mougias A., Christidi F., Kaldi M., Kerossi M.-I., Athanasouli P., Politis A. (2020). Mini-Mental State Examination: Greek Normative Data Stratified by Age and Education in a Large Sample of 925 Community-Dwelling Healthy Participants. Adv. Exp. Med. Biol..

[37] Partington J.E., Leiter R.G. (1949). Partington’s Pathways Test. Psychol. Serv. Cent. J..

[38] Zalonis I., Kararizou E., Triantafyllou N.I., Kapaki E., Papageorgiou S., Sgouropoulos P., Vassilopoulos D. (2008). A Normative Study of the Trail Making Test A and B in Greek Adults. Clin. Neuropsychol..

[39] Βλάχου Χ.-Ε., Κοσμίδου Μ.-Ε. (2002). H ελληνική Δοκιμασία Oπτικό-Νοητικής Ιχνηλάτησης: Προκαταρκτικές νόρμες για κλινική και ερευνητική εφαρμογή. Psychol. J. Hell. Psychol. Soc..

[40] Lezak M.D., Howieson D.B., Bigler E.D., Tranel D., Lezak M.D., Howieson D.B., Bigler E.D., Tranel D. (2012). Neuropsychological Assessment.

[41] Tsiakiri A., Christidi F., Tsiptsios D., Vlotinou P., Kitmeridou S., Bebeletsi P., Kokkotis C., Serdari A., Tsamakis K., Aggelousis N. (2024). Processing Speed and Attentional Shift/Mental Flexibility in Patients with Stroke: A Comprehensive Review on the Trail Making Test in Stroke Studies. Neurol. Int..

[42] Patricacou A., Psallida E., Pring T., Dipper L. (2007). The Boston Naming Test in Greek: Normative Data and the Effects of Age and Education on Naming. Aphasiology.

[43] Roth C., Kreutzer J.S., DeLuca J., Caplan B. (2011). Boston Naming Test. Encyclopedia of Clinical Neuropsychology.

[44] Kosmidis M.H., Vlahou C.H., Panagiotaki P., Kiosseoglou G. (2004). The Verbal Fluency Task in the Greek Population: Normative Data, and Clustering and Switching Strategies. J. Int. Neuropsychol. Soc..

[45] Kounti F., Tsolaki M., Kiosseoglou G. (2006). Functional Cognitive Assessment Scale (FUCAS): A New Scale to Assess Executive Cognitive Function in Daily Life Activities in Patients with Dementia and Mild Cognitive Impairment. Hum. Psychopharmacol..

[46] Hamilton M. (1960). A Rating Scale for Depression. J. Neurol. Neurosurg. Psychiatry.

[47] Bandyopadhyay S., Ganguli B., Chatterjee A. (2011). A Review of Multivariate Longitudinal Data Analysis. Stat. Methods Med. Res..

[48] Ottenbacher K.J. (1998). Quantitative Evaluation of Multiplicity in Epidemiology and Public Health Research. Am. J. Epidemiol..

[49] Pendlebury S.T., Mariz J., Bull L., Mehta Z., Rothwell P.M. (2012). MoCA, ACE-R, and MMSE versus the National Institute of Neurological Disorders and Stroke-Canadian Stroke Network Vascular Cognitive Impairment Harmonization Standards Neuropsychological Battery after TIA and Stroke. Stroke.

[50] Wiberg B., Lind L., Kilander L., Zethelius B., Sundelöf J.E., Sundström J. (2010). Cognitive Function and Risk of Stroke in Elderly Men. Neurology.

[51] Fishman K.N., Ashbaugh A.R., Lanctôt K.L., Cayley M.L., Herrmann N., Murray B.J., Sicard M., Lien K., Sahlas D.J., Swartz R.H. (2018). Apathy, Not Depressive Symptoms, as a Predictor of Semantic and Phonemic Fluency Task Performance in Stroke and Transient Ischemic Attack. J. Clin. Exp. Neuropsychol..

[52] Brady C.B., Spiro A., McGlinchey-Berroth R., Milberg W., Gaziano J.M. (2001). Stroke Risk Predicts Verbal Fluency Decline in Healthy Older Men: Evidence from the Normative Aging Study. J. Gerontol. B Psychol. Sci. Soc. Sci..

[53] Volz M., Ladwig S., Werheid K. (2021). Gender Differences in Post-Stroke Depression: A Longitudinal Analysis of Prevalence, Persistence and Predictive Value of Known Risk Factors. Neuropsychol. Rehabil..

[54] Dong L., Sánchez B.N., Skolarus L.E., Stulberg E., Morgenstern L.B., Lisabeth L.D. (2020). Sex Difference in Prevalence of Depression after Stroke. Neurology.

[55] Mayman N.A., Tuhrim S., Jette N., Dhamoon M.S., Stein L.K. (2021). Sex Differences in Post-Stroke Depression in the Elderly. J. Stroke Cerebrovasc. Dis..

[56] Roivainen E. (2011). Gender Differences in Processing Speed: A Review of Recent Research. Learn. Individ. Differ..

[57] Horstmann S., Rizos T., Rauch G., Arden C., Veltkamp R. (2014). Feasibility of the Montreal Cognitive Assessment in Acute Stroke Patients. Eur. J. Neurol..

[58] Pasi M., Salvadori E., Poggesi A., Inzitari D., Pantoni L. (2013). Factors Predicting the Montreal Cognitive Assessment (MoCA) Applicability and Performances in a Stroke Unit. J. Neurol..

[59] Cova I., Mele F., Zerini F., Maggiore L., Cucumo V., Brambilla M., Rosa S., Bertora P., Salvadori E., Pomati S. (2020). Neuropsychological Screening in the Acute Phase of Cerebrovascular Diseases. Acta Neurol. Scand..

[60] Mainz N., Shao Z., Brysbaert M., Meyer A.S. (2017). Vocabulary Knowledge Predicts Lexical Processing: Evidence from a Group of Participants with Diverse Educational Backgrounds. Front. Psychol..

[61] Christidi F., Kararizou E., Triantafyllou N., Anagnostouli M., Zalonis I. (2015). Derived Trail Making Test Indices: Demographics and Cognitive Background Variables across the Adult Life Span. Aging Neuropsychol. Cogn..

[62] Wiberg B., Kilander L., Sundström J., Byberg L., Lind L. (2012). The Relationship between Executive Dysfunction and Post-Stroke Mortality: A Population-Based Cohort Study. BMJ Open.

[63] Tamez E., Myerson J., Morris L., White D.A., Baum C., Connor L.T. (2011). Assessing Executive Abilities Following Acute Stroke with the Trail Making Test and Digit Span. Behav. Neurol..

[64] Wouters A., Nysten C., Thijs V., Lemmens R. (2018). Prediction of Outcome in Patients With Acute Ischemic Stroke Based on Initial Severity and Improvement in the First 24 h. Front. Neurol..

[65] Clancy U., Makin S.D.J., McHutchison C.A., Cvoro V., Chappell F.M., del Hernández M.C.V., Sakka E., Doubal F., Wardlaw J.M. (2022). Impact of Small Vessel Disease Progression on Long-Term Cognitive and Functional Changes After Stroke. Neurology.

[66] Tilling K., Sterne J.A.C., Rudd A.G., Glass T.A., Wityk R.J., Wolfe C.D.A. (2001). A New Method for Predicting Recovery After Stroke. Stroke.

[67] Tsiakiri A., Vlotinou P., Terzoudi A., Heliopoulos I., Vadikolias K. (2022). Cognitive, Functional, and Emotional Changes During the COVID-19 Pandemic in Greek Patients with Neurocognitive Disorders. J. Alzheimers Dis..

[68] Nakao S., Takata S., Uemura H., Kashihara M., Osawa T., Komatsu K., Masuda Y., Okahisa T., Nishikawa K., Kondo S. (2010). Relationship between Barthel Index Scores during the Acute Phase of Rehabilitation and Subsequent ADL in Stroke Patients. J. Med. Investig..

[69] Lin J.-H., Lin R.-T., Tai C.-T., Hsieh C.-L., Hsiao S.-F., Liu C.-K. (2003). Prediction of Poststroke Dementia. Neurology.

[70] Lim J.-S., Oh M.S., Lee J.-H., Jung S., Kim C., Jang M.U., Lee S.-H., Kim Y.J., Kim Y., Park J. (2017). Prediction of Post-Stroke Dementia Using NINDS-CSN 5-Minute Neuropsychology Protocol in Acute Stroke. Int. Psychogeriatr..

[71] Dienanta S.B., Hamdan M., Soetjipto S., Machin A. (2020). The Relevance of Right and Left Hemisphere Classification to Predict Cognitive Outcome After Stroke. J. Indones. Med. Assoc..

[72] Fink J.N., Frampton C.M., Lyden P., Lees K.R. (2008). Does Hemispheric Lateralization Influence Functional and Cardiovascular Outcomes After Stroke?. Stroke.

[73] Almekhlafi M.A., Hill M.D., Roos Y.M., Campbell B.C.V., Muir K.W., Demchuk A.M., Bracard S., Gomis M., Guillemin F., Jovin T.G. (2019). Stroke Laterality Did Not Modify Outcomes in the HERMES Meta-Analysis of Individual Patient Data of 7 Trials. Stroke.

